# Comparative metagenomic analysis of gut microbiota in *Anacanthotermes turkestanicus* and *A. ahngerianus* reveals diet- and habitat-driven functional divergence

**DOI:** 10.3389/finsc.2026.1807673

**Published:** 2026-07-14

**Authors:** Ulugbek Togaev, Vartika Mathur, Aziza Rakhmonkulova, Surbhi Agarwal, Aarav Mathur, Shuhrat Turageldiyev, Rasul Ruzmetov, Abboskhon S. Turaev, Zaitjan Tillyabaev, Alimjan Matchanov, David Sillam-Dussès

**Affiliations:** 1 Institute of Bioorganic Chemistry, Academy of Science of Uzbekistan, Tashkent, Uzbekistan; 2 Engineering Faculty, Central Asian University (CAU) Central Asian University in Tashkent, Tashkent, Uzbekistan; 3 Animal-Plant Interactions Lab, Department of Zoology, Sri Venkateswara College, New Delhi, India; 4 Max Planck Institute for Chemical Ecology, Jena, Germany; 5 Department of Agronomy, Urganch State University, Urganch, Uzbekistan; 6 Laboratory of Experimental and Comparative Ethology, Laboratory of Experimental and Comparative Ethology (LEEC), Unité de Recherche (UR) 4443, University Sorbonne Paris Nord, Villetaneuse, France

**Keywords:** archaea, bacteria, lignocellulose, metagenome, microbes, shotgun sequencing, termites

## Abstract

The gut microbiome of termites plays a crucial role in lignocellulose degradation and nutrient recycling. This study presents the first metagenomic characterization of the gut microbiota in two lower termite species, *Anacanthotermes ahngerianus* and *Anacanthotermes turkestanicus*, collected from distinct ecological habitats. In Uzbekistan, the first lives in building a mound in nature in the West part while the second mainly lives in contact with human constructions in the East part without building a proper mound. Both species showed similar bacterial dominance (~53%) in their guts but *A. ahngerianus* exhibited higher overall microbial diversity (Shannon index: 4.046 vs. 3.363; Simpson’s index: 0.927 vs. 0.776). Moreover, both termite species showed differences in microbial profiles, including bacterial taxa and eukaryotic groups relevant to lower-termite gut symbiosis. Protist-associated eukaryotic reads were retained because flagellated protists are essential symbionts of lower termites, whereas unexpected non-protist eukaryotic assignments were interpreted cautiously and were not used as evidence of functional gut symbionts or host adaptation. Functional profiling revealed enrichment of pathways related to carbohydrate metabolism, amino acid transport, and energy production in both species. However, *A. turkestanicus* exhibited stronger bacterial dominance associated with lignocellulose degradation and nitrogen cycling, while *A. ahngerianus* maintained a more balanced representation of bacteria, fungi, and viruses. These findings suggest that species identity and ecological habits may be associated with differences in gut microbiome structure and predicted functional potential.

## 1 Introduction

Gut microbiome of termites has been a subject of immense interest due to its crucial role in the digestion of lignocellulosic materials, the primary food source for these insects. Lignocellulose, the predominant constituent of the plant cell wall, is composed of an intricate matrix of lignin, cellulose, and hemicellulose, and is typically resistant to direct digestion by animals. Although certain insects, such as higher termites and xylophagous cockroaches, have evolved endogenous lignocellulolytic enzymes (1, 2), various taxa of lignocellulose-consuming animals, especially insects, harbor specialized gut microbiota that facilitate lignocellulose degradation (3). A notable instance of this symbiotic relationship is observed in termites (4, 5). These social insects are conventionally divided into lower termites (all families excluding Termitidae) and higher termites (family Termitidae), the latter representing nearly 75% of all termite species (6). Higher termites lack protist symbionts and primarily rely on gut bacteria and, in the family Macrotermitinae, on external fungal associations to decompose lignocellulose. In contrast, the hindgut of lower termites harbors a dense community of eukaryotic symbionts (flagellated protists) that function synergistically with gut bacteria (5, 7, 8). This microbial community forms complex symbiotic relationships that enable the efficient degradation of lignocellulose (9). This intricate relationship between termites and their associated microbes, collectively known as the termite holobiont, has been a central focus of research for decades (1, 10, 11). This is widely recognized that the symbiotic microbial communities with termites are essential for the digestion of lignocellulose, as well as for their host’s immunity, reproduction, and various other physiological functions (5, 12–15). Interestingly, the gut microbiome exhibits a high degree of specificity, aligning more strongly with the foraging and wood-dwelling ecologies of the host species rather than their phylogenetic relatedness (16, 17). This suggests that environmental factors and dietary preferences, rather than solely evolutionary history, shaped the termite gut microbiome. The ability of termites to efficiently process lignocellulose makes them a keystone taxon in the global carbon cycle, and understanding the detailed functions of each microbial species within their gut is crucial for elucidating this process (18). Although termites play a crucial beneficial role in natural ecosystem by transforming organic matter and significantly modifying the soil environment through their mechanical activities (12, 19, 20), approximately two hundred termite species are known to damage buildings, crops, pastures, forestry, and non-cellulose materials such as electrical cables. The global damage caused by them and subsequent repair costs are estimated at $40 billion annually (21–23). They damage crops and wreak havoc on wooden structures, ranging from homes to heritage buildings. The impact is particularly pronounced in regions like Uzbekistan, where termites belonging to *Anacanthotermes turkestanicus* have caused more than a million in damages to cultural heritage sites in the last 30 years alone (24). For instance, the “Ichan-Kala” historical complex, a UNESCO-protected site, faces a considerable challenge as termites damage the detailed wooden carvings on 33 historical monuments (23). The problem of termite damage is particularly acute in the Republic of Karakalpakstan, Khorezm, Kashkadarya, Surkhandarya, Navoiy, and Samarkand regions. Conventional control of pest insects that damage buildings and crops has mainly relied on chemical insecticides; however, these compounds may have negative effects on non-target organisms and may contribute to resistance development and reduced efficacy over time. In this context, understanding termite biology, especially the role of gut microorganisms in digestion, nutrient acquisition, and environmental adaptation, is important for developing more targeted and sustainable control strategies. Since termite gut bacteria participate in lignocellulose degradation and host homeostasis, the microbial profiles observed in the present study may provide baseline information for future approaches aimed at disrupting essential digestive or physiological processes in pest termites. Therefore, studying gut microbiota in termite species that are economically important in Uzbekistan.

Emerging strategies in pest control emphasize sustainability, automation, and precision. For example, intelligent monitoring systems using wireless sensor networks, real-time data processing, and GPS positioning have been developed to detect and control termite activity efficiently in hydrological infrastructures (25–27). In parallel, understanding the termite gut microbiome has become increasingly important, as the digestion of lignocellulose in lower termites relies on a complex consortium of symbiotic bacteria and protists. These microbial symbionts enzymatically hydrolyze cellulose and hemicellulose into fermentable monosaccharides, with specific taxa such as *Treponema* and *Fibrobacteres*, and protists like *Trichonympha*, playing essential roles in these biochemical processes (28). Because lower termites strongly depend on their gut flagellates for lignocellulose digestion, this microbiome-centered insight offers novel avenues for targeted, microbiota-based termite control strategies. Therefore, lower termites provide a useful system for understanding how gut flagellates support termite survival, digestion, and ecological success. Microbial symbiosis directly influences termite metabolic pathways and nutrient assimilation by enhancing the breakdown of lignocellulosic material, thereby improving the efficiency of energy extraction from their cellulose-rich diet—an essential process for their survival, development, and ecological success. In addition to their contributions to digestion, these microorganisms may provide protection for termites against pathogens but also participate in the nutrient cycling within their environment, highlighting their multifaceted roles in both host health and ecosystem functioning (11, 29).

Previous investigations of termite gut microbiomes have predominantly relied on targeted amplicon sequencing of the 16S rRNA gene, which, while valuable for bacterial community profiling, is inherently limited to prokaryotic domains and is subject to primer bias that may systematically under- or over-represent certain taxa (30). Moreover, amplicon-based approaches provide no direct functional information, precluding assessment of metabolic pathways, lignocellulolytic gene repertoires, or cross-kingdom interactions that are central to termite digestive physiology (31). Shotgun metagenomic sequencing addresses these limitations by enabling simultaneous, unbiased profiling of all microbial groups including bacteria, archaea, eukaryota, and viruses alongside reconstruction of functional potential from community-level genomic data. For example, Marynowska et al. (29) characterized biomass-degrading communities in higher termites, while Arora et al. (32) investigated the evolutionary diversification of termite gut functions across multiple termite lineages. More recently, Xie et al. (33) and Dar et al. (31) demonstrated substantial variation in microbial metabolic pathways among termites with different feeding strategies. Despite these advances, lower termites from Central Asia remain almost entirely unexplored. This multi-kingdom resolution is particularly critical for lower termites such as *Anacanthotermes* species, whose hindgut ecosystem is defined by intimate syntrophic interactions among flagellated protists, methanogenic archaea, and diverse bacterial lineages that cannot be captured by any single-marker approach.

In this study, we aim to investigate the gut microbiome of two lower termite species, *A. turkestanicus* and *A. ahngerianus* (Order: Blattodea; Family: Hodotermitidae), and explore the potential functions of their host–microsymbiont interactions. In Uzbekistan, *A. turkestanicus* is the most widespread and economically important termite species, particularly in arid and semi-arid regions (34, 35). *A. ahngerianus* occurs in desert and semi-desert landscapes, including areas of the Kyzylkum and Ustyurt deserts, where they construct subterranean tunnel systems and galleries (24) that facilitate foraging under dry environmental conditions. *A. ahngerianus* feeds mainly on dry plant material, including grasses, stems, and woody substrates (23, 35), while *A. turkestanicus* mainly infests wooden structures and historically important buildings. Previous studies have reported its destructive impact on residential buildings and cultural heritage monuments in Uzbekistan (34). Accordingly, these species represent suitable models for investigating how gut microbial communities may be associated with ecological adaptation and lignocellulose utilization.

Although gut microbiomes of termites have been widely studied, Central Asian lower termites remain poorly represented in comparative metagenomic research. In particular, no shotgun metagenomic comparison has previously been conducted for *Anacanthotermes turkestanicus* and *A. ahngerianus*, two closely related termite species from Uzbekistan. Because both species belong to *Hodotermitidae* and are expected to share conserved lower-termite symbiotic features, including associations with bacteria, archaea, and flagellated protists, we do not assume that their gut microbiomes are entirely distinct from those of previously studied lower termites. Rather, this study adds regional and taxonomic coverage to the broader understanding of lower-termite gut microbiomes and provides the first metagenomic baseline for *Anacanthotermes* species from Central Asian arid and semi-arid ecosystems. *A. turkestanicus* was collected from an urban wood-infested environment, whereas *A. ahngerianus* was collected from natural nest material in an arid/semi-arid landscape. Although both species share broadly similar lignocellulosic feeding preferences, these different collection contexts may expose termite workers to different nesting substrates, soil-associated microorganisms, environmental DNA, plant residues, anthropogenic materials, and microclimatic conditions. Therefore, we interpret potential microbiome differences as ecological-origin-associated patterns rather than as direct evidence of diet-driven divergence. We used shotgun metagenomic sequencing to compare the taxonomic composition, alpha diversity, and predicted functional potential of the gut microbiomes of these two termite species. We hypothesized that, despite their close taxonomic relationship and shared lower-termite symbiotic framework, *A. turkestanicus* and *A. ahngerianus* may show differences in gut microbial composition and predicted functional profiles associated with ecological origin. This study provides a baseline for future replicated sampling, controlled feeding experiments, metatranscriptomics, metabolomics, and metagenome-assembled genome analyses of Central Asian desert termites. We further hypothesized that these microbial differences are reflected in predicted functional pathways related to lignocellulose degradation, carbohydrate metabolism, nutrient acquisition, and environmental adaptation. By testing this hypothesis, the present study provides a baseline for understanding termite–microbiome interactions in Central Asian desert termites and may serve as a reference for future studies on termite ecology, lignocellulose bioconversion, and microbiome-based termite management strategies.

## 2 Materials and methods

### 2.1 Sample collection

**Figure 1:**
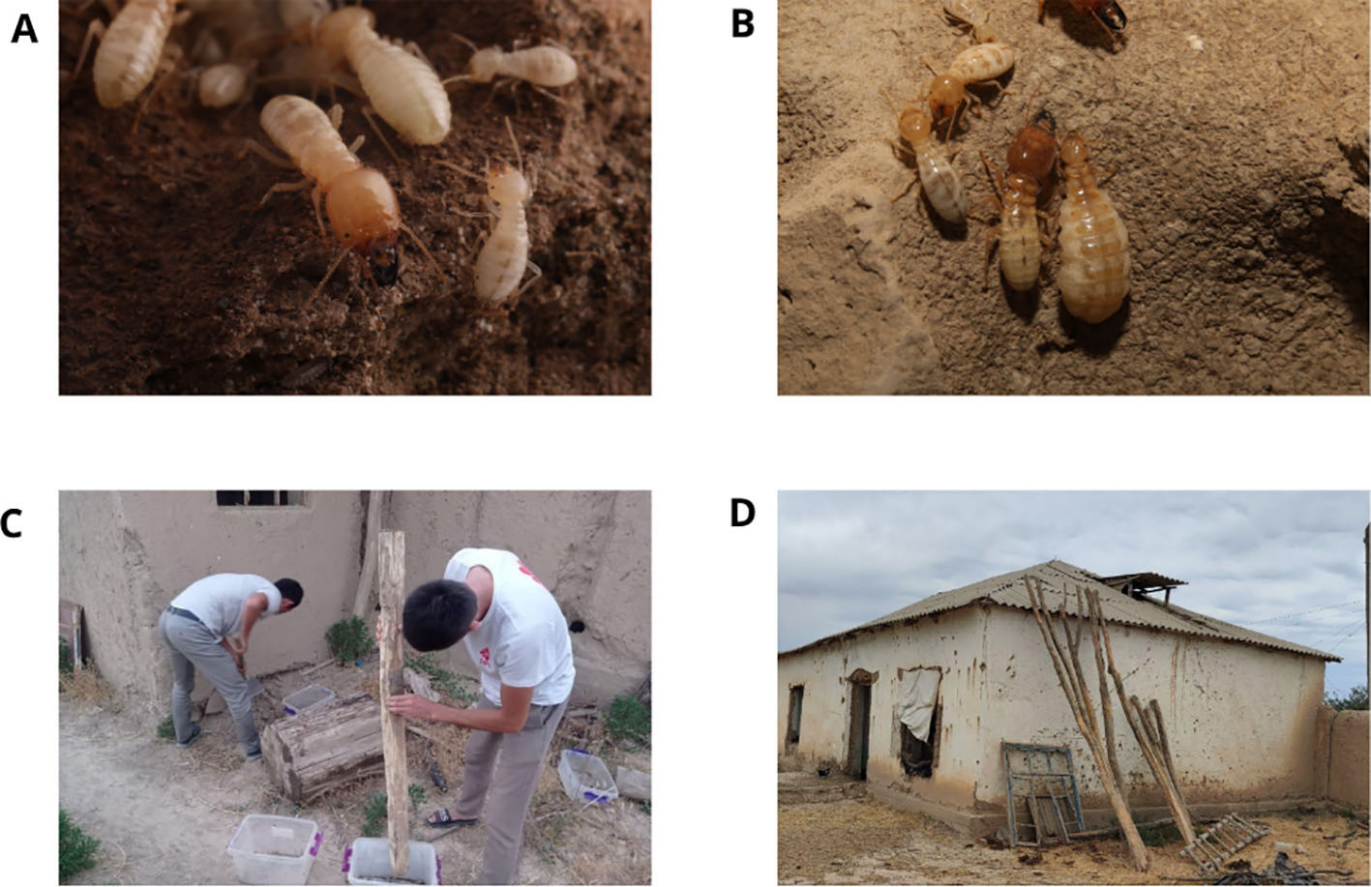
Morphology, distribution, and damage of *Anacanthotermes* species. **(A)** Workers and soldiers in *A. ahngerianus* (Credit: Jan Šobotník). **(B)** Workers in *A. turkestanicus* (Credit: Jan Šobotník). **(C)** Collection localities for *Anacanthotermes* species. **(D)** Termite damage observed at collection sites.

Workers of the termite *A. ahngerianus* (**Figure 1A**) were collected from one of the nests they built in Khiva District (41.40476° N, 60.29471° E), Xorazm Region, Uzbekistan. *A. turkestanicus* workers (**Figure 1B**) were collected from damaged houses in Chinar village (40.2793010° N, 65.882021° E), Xatirchi District, Navoiy region, Uzbekistan. Field collection sites and representative termite damage are illustrated in **Figures 1C, D**. Collected termites were maintained in the laboratory at 26 ± 2°C and 95% RH in a plastic container containing a moist filter paper.

### 2.2 Dissection of termite gut

For each termite species, 15 fifth-instar worker termites were selected for gut dissection. These 15 individuals represented one pooled biological sample per species and were used to obtain sufficient gut-derived DNA for shotgun metagenomic sequencing. Prior to dissection, termite workers were briefly rinsed with sterile distilled water to remove external debris. Dissections were performed under aseptic conditions using sterilized fine-tip forceps and scissors. All instruments were sterilized before use and between samples using 70% ethanol, followed by drying under sterile conditions. The dissection surface was cleaned with 70% ethanol, and gut removal was carried out under a clean working environment to minimize external microbial contamination. The whole gut was carefully pulled out from the abdomen of each worker using sterilized forceps. Dissected guts from 15 workers of the same termite species were pooled into a sterile 1.5 mL microcentrifuge tube containing RNAlater™ Stabilization Solution (Thermo Fisher Scientific, USA). Samples were placed on ice during processing and subsequently stored at −20 °C until DNA extraction. The pooled gut tissue sample was transferred to a sterile 1.5 mL microcentrifuge tube and minced in sterile phosphate-buffered saline (PBS, pH 7.2) using sterile fine-tip forceps before DNA extraction. Genomic DNA was then isolated using the Qiagen DNeasy^®^ Blood & Tissue Kit (Hilden, Germany) according to the manufacturer’s instructions.

### 2.3 DNA extraction and purification

Guts of the 15 workers were pooled into a single composite sample for each species. DNA was isolated using the Qiagen DNeasy® Blood & Tissue Kit (Hilden, Germany) according to the manufacturer’s instructions. A gut tissue sample (~0.025 g) was excised, minced, and placed in a 1.5 mL microcentrifuge tube. The sample underwent a series of buffer treatments and centrifugation steps to obtain high-quality genomic DNA. Concentration, quality and purity of DNA were analyzed using gel electrophoresis and NanoDrop ND1000 (NanoDrop Technologies Inc., Wilmington, USA). The A260/A280 ratio was found to be in the range of ~1.8–2.0, indicating high-quality DNA.

### 2.4 Library preparation and sequencing

Enzymatic DNA fragmentation and library preparation were performed using the KAPA HyperPlus Kit (Kapa Biosystems, Cape Town, South Africa). Extracted DNA was fragmented into ~ 600 bp lengths using KAPA Frag Kit (Kapa Biosystems, Cape Town, South Africa). The fragmented samples were processed for end repair and A-tailing with HyperPlus ERAT enzyme mix. Immediately after the end repair and A-tailing, the adapters were added and ligated to the end repaired DNA fragments using DNA ligase. Library amplification was done to the adapter-ligated samples with Illumina primers. Libraries were evaluated by gel-electrophoresis. Extracted DNA (40 ng) was used for amplification along with 10 pM of each primer. PCR was performed following program as follows: 45 s of initial denaturation at 98 °C followed by 4 cycles of denaturation (15 s at 98 °C), annealing (30 s at 60 °C) and extension (30 s at 72 °C) with final extension at 72 °C for 1 min. Libraries were purified using Agencourt AMPure XP (Beckman Coulter Inc., CA, USA). Final concentration was measured on Qubit dsDNA High Sensitivity Assay Kit (Thermo Fisher Scientific, Waltham, MA, USA) on Qubit Fluorometer (Life Technologies, Carlsbad, CA, USA). Product size selection for sequencing was based on 0.7x (>450 bp). Sequencing was performed using Illumina HiSeq 4000 (Illumina, San Diego, CA, USA).

### 2.5 Bioinformatics pipeline and quality control

Raw sequencing reads were assessed for quality using FastQC (v0.12.0) (36). Adapter trimming, quality filtering, and removal of low-quality reads was performed using fastp (v1.3.3) (37) with default parameters to remove low-quality reads. To remove potential laboratory-derived contamination, reads were aligned against the human reference genome (GRCh38/hg38) using Bowtie2 (v2.4.5) (38), and reads mapping to the human genome were excluded from downstream analysis.

### 2.6 Taxonomic profiling and alpha diversity analysis

As reference genomes for *Anacanthotermes ahngerianus* and *A. turkestanicus* are currently unavailable, host-genome depletion via alignment was not performed for the termite host. Instead, taxonomic classification of the quality-filtered reads was carried out using Kraken2 (v2.1.3) (39) against the core_nt database (v2024.09). This k-mer-based approach allowed for the simultaneous identification of microbial taxa and the separation of microbial sequences from host-derived eukaryotic reads. Kraken2 classification results were further processed to retain only microbial sequences (Bacteria, Archaea, Viruses, and Eukaryota) for downstream analysis.

Species abundance was calculated using Bracken (v3.1) (40). To evaluate the complexity of gut microbial communities, alpha diversity indices were calculated from Bracken-estimated species-level abundance profiles using KrakenTools (v1.2) (41) implemented within the Galaxy EU (42) platform. Bracken redistributes reads initially classified at higher taxonomic ranks to the species level using a probabilistic model trained on the reference database. Shannon’s diversity index (H’) and Simpson’s index of diversity (1−D) were calculated to assess community diversity incorporating both species richness and the evenness of abundance distribution. The Berger-Parker index was computed to quantify the degree of dominance by the most abundant taxon. Fisher’s alpha was additionally calculated as a parametric richness estimator that assumes a log-series abundance distribution and is robust to variation in total read depth between samples.

### 2.7 Functional annotation and data visualization

Functional annotation of the classified reads was performed using COGclassifier against the Clusters of Orthologous Groups (COGs) database (43). The COG database was selected because it provides broad orthologous gene categories that are useful for summarizing the predicted functional potential of metagenomic communities, including functions related to carbohydrate metabolism, amino acid metabolism, energy production, replication and repair, and defense mechanisms. Pathway prediction was performed using GhostKOALA (44) against the Kyoto Encyclopedia of Genes and Genomes (KEGG) database (45), because KEGG-based annotation enables the prediction of metabolic pathways and provides complementary information to COG-based functional classification. Classification results were visualized as Sankey plots using Pavian (v1.0) (46), which allows interactive exploration and visualization of metagenomic taxonomic profiles. Summary statistics across all samples were aggregated and visualized using MultiQC (v1.33.1) (47). Additional figures including taxonomic composition charts were generated using Python (v3.10.12) (48) with the pandas (v2.3.0) (49) and Plotly (v6.7.0) libraries for data organization and processing while Plotly was used to generate clear visual representations of taxonomic and functional patterns. As each termite species was represented by one pooled gut sample consisting of 15 workers, the functional profiles were interpreted descriptively and with caution. Therefore, observed differences in COG categories and KEGG-predicted pathways were considered indicators of predicted functional potential rather than statistically confirmed functional differences between species.

## 3 Results

### 3.1 Sequencing data and read counts

Approximately 36.5 million reads were generated for *A. ahngerianus* and 29.7 million reads for *A. turkestanicus*, with an average read length of 150 base pairs. Quality filtering with fastp showed that the majority of reads were high quality, with over 99.9% passing the initial filters and Q30 scores above 85%. After quality control and trimming, as well as the removal of potential human contamination, 924,255 reads for *A. ahngerianus* and 587,978reads for *A. turkestanicus* were taxonomically classified using the Kraken2 core_nt database.

### 3.2 Comparative analysis of gut microbiota

**Figure 2:**
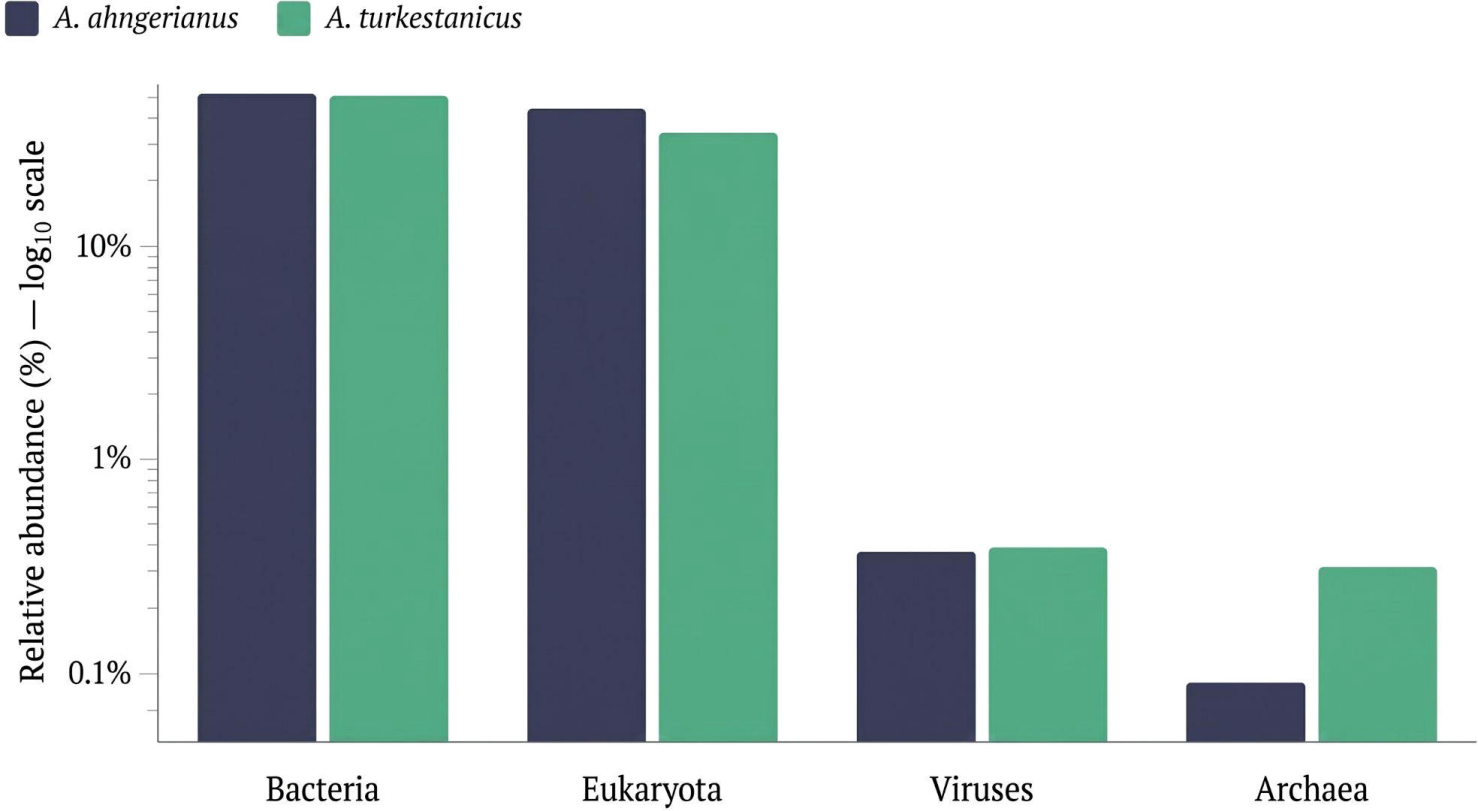
Relative abundance of four microbial kingdoms in the termites *A. ahngerianus* and *A. turkestanicus.*.

The metagenomic analysis revealed that both termite species harbored diverse microbial communities, including members from bacteria, archaea, eukaryota (fungi and protozoa), and viruses. **Figure 2** compares microbial diversity associated with *A. ahngerianus* and *A. turkestanicus*.

**Figure 3:**
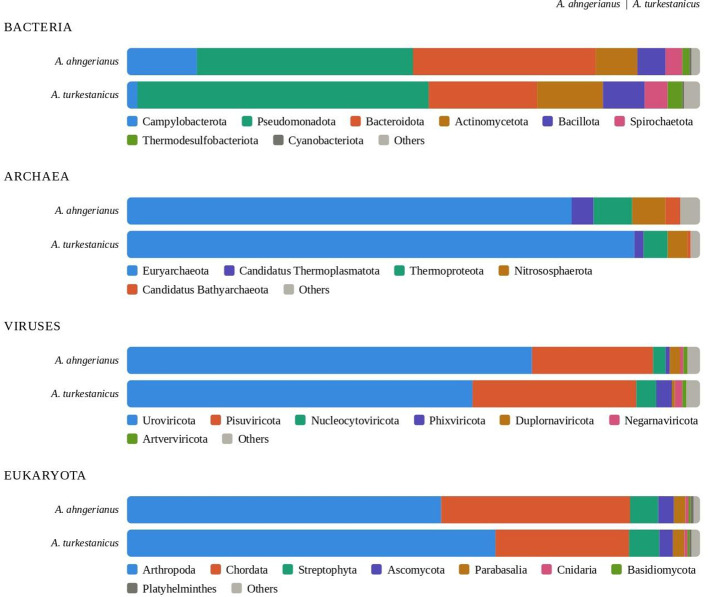
Phylum-level relative abundance of classified reads within each microbial kingdom in both termite species.

**Figure 4:**
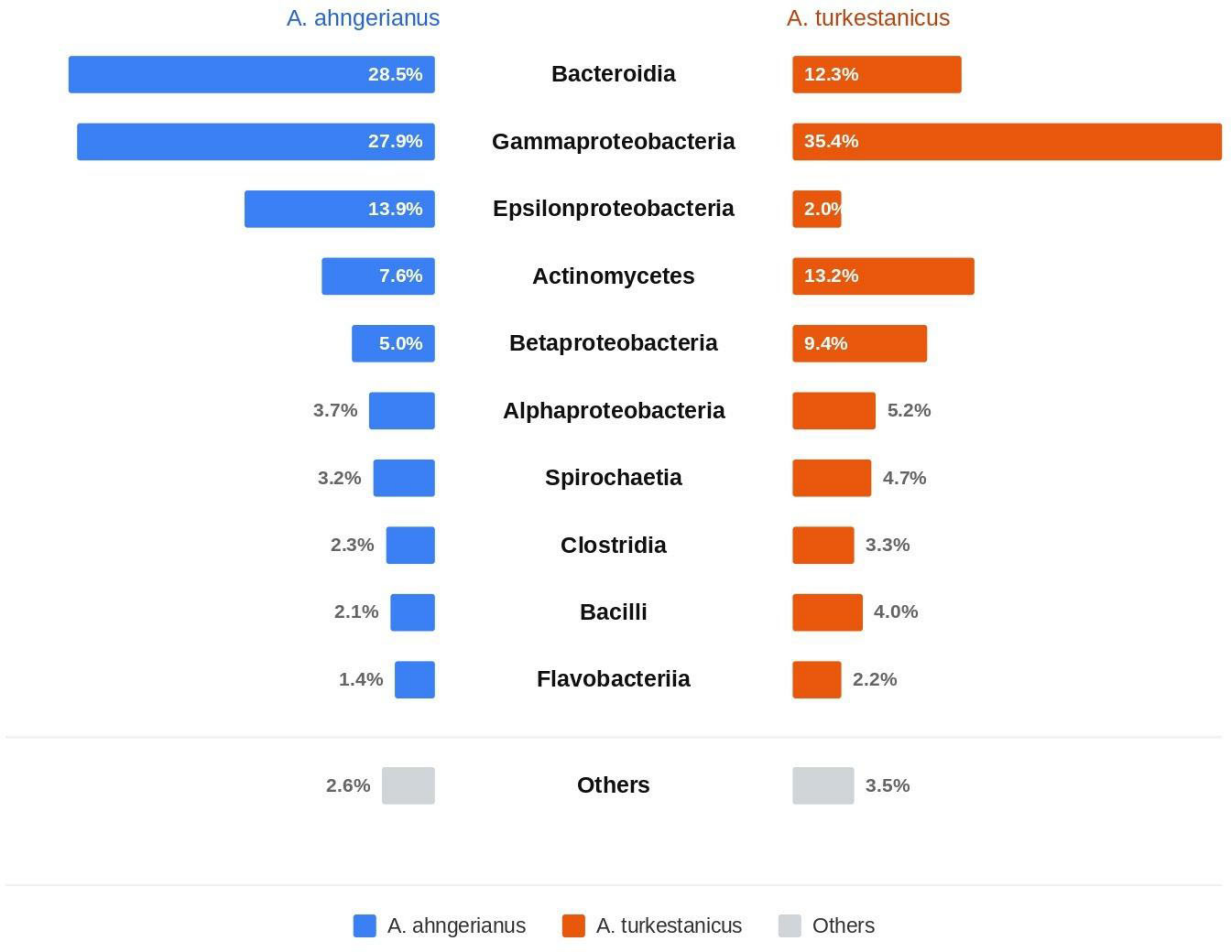
Relative abundance of bacterial classes in *A. turkestanicus* and *A. ahngerianus*.

The most abundant bacterial phyla across both species were Pseudomonadota, Bacteroidota, Actinomycetota, Bacillota, Spirochaetota, and Campylobacterota (**Figure 3**). Both termite species exhibited differences in microbial composition at the class and family levels. The microbial communities showed similar relative abundance of bacteria both in to *A. ahngerianus* and *A. turkestanicus* (53.66% vs. 52.98%, respectively, **Table 1**). Classes such as Gammaproteobacteria, Actinomycetes, Betaproteobacteria, Alphaproteobacteria, and Spirochaetia were found in greater abundance in *A. turkestanicus* than in *A. ahngerianus* (**Figure 4**). At the genus level, we observed notable differences in the microbial taxa between *A. turkestanicus* and *A. ahngerianus*. Certain bacterial and eukaryotic genera, such as *Ruficoccus*, *Noditermes*, and *Dicholtrachelus*, were unique to *A. turkestanicus*, while genera like *Rhinotermes, and Rhiginia* were exclusively found in *A. ahngerianus* (**Table 2**).

**Table 1:** Relative abundance and raw read counts by groups for the gut microbiomes of *A. ahngerianus* and *A. turkestanicus*.

Groups	Reads *A. ahngerianus*	Reads *A. turkestanicus*	Abundance (%) *A. ahngerianus*	Abundance (%) *A. turkestanicus*
Bacteria	495,948	311,489	53.66	52.98
Eukaryota	424,030	272,351	45.88	46.32
Viruses	3,421	2,281	0.3701	0.3879
Archaea	856	1,857	0.0926	0.3158
**Total**	**924,255**	**587,978**	**100.00**	**100.00**

**Table 2:** Microbial genera exclusively found in *A. ahngerianus* and *A. turkestanicus* (+, presence; -, absence).

Genus	*A. ahngerianus*	*A. turkestanicus*	Kingdom
*Rhinotermes*	+	–	Eukaryota
*Sphodromantis*	+	–	Eukaryota
*Cryptophyllium*	+	–	Eukaryota
*Diaphanes*	+	–	Eukaryota
*Coomania*	+	–	Eukaryota
*Rhiginia*	+	–	Eukaryota
*Churamiti*	+	–	Eukaryota
*Dieffenbachia*	+	–	Eukaryota
*Trichomitopsis*	+	–	Eukaryota
*Ruficoccus*	–	+	Bacteria
*Noditermes*	–	+	Eukaryota
*Dichotrachelus*	–	+	Eukaryota

Comparative analysis of eukaryotic gut microbiota revealed broadly similar community profiles between the two termite species. Among fungal phyla, Ascomycota and Basidiomycota were detected in both species. Notably, Parabasalia — a phylum comprising flagellated protists central to lignocellulose digestion in lower termites — was detected in both species, consistent with their classification as lower termites. These protists belong to the phylum Parabasalia or the order Oxymonadida and are transmitted between colony members via proctodeal trophallaxis, contributing to patterns of co-speciation between protist and termite host (17). Their detection here confirms the tripartite symbiotic system expected in *Anacanthotermes* species. Recent phylogenetic analyses of Anacanthotermes-associated parabasalids (34) demonstrated a long evolutionary history of host-symbiont associations and revealed several symbiont lineages unique to this termite genus. Our metagenomic results support these observations and confirm that the tripartite symbiotic system remains a central component of digestive functioning in Anacanthotermes. Streptophyta reads, likely reflecting plant-derived dietary DNA retained in the gut, were also present in both species, consistent with their lignocellulosic diet (50).

Interestingly, although the sequence count for viruses was higher in *A. ahngerianus* than *A. turkestanicus*, the gut metagenome of *A. turkestanicus* exhibited a more diverse viral population. The phylum Uroviricota was predominant in both termite species (more than 60% of total viral sequences, **Figure 3**), but *A. turkestanicus* showed a greater relative richness in viral taxa, suggesting a more complex viral ecosystem. This suggests bacterial and viral dominance with minimal variation in archaea, fungi, and protozoa between these termite species. The archaea were the least abundant of all communities and dominated by Euryarchaeota, with smaller proportions of Thermoproteota, Nitrososphaerota, Candidatus Thermoplasmatota and Candidatus Bathyarchaeota across both termite species.

### 3.3 Alpha diversity analysis

Across all four indices, *A. ahngerianus* consistently shows higher gut microbiome diversity than *A. turkestanicus*, and the indices are internally coherent. Shannon’s H’ (4.046 vs 3.363) and Simpson’s 1−D (0.927 vs 0.776) both reflect that *A. ahngerianus* harbors a more even, species-rich community (**Table 3**). The key contrast comes from the Berger-Parker index: *A. turkestanicus* scores 0.465, meaning nearly half of all reads are assigned to a single dominant taxon — in this case Gammaproteobacteria (35.4% at class level). *A. ahngerianus*, with a Berger-Parker of 0.204, is less dominated by any single taxon, consistent with Bacteroidia (28.5%) and Gammaproteobacteria (27.9%) co-dominating more evenly (**Figure 4**). Fisher’s alpha (240 vs 214) agrees with the same trend, with the difference being more modest because Fisher’s alpha is less sensitive to dominance. The high absolute Fisher’s alpha values in both samples are expected for shotgun metagenomics — unlike 16S amplicon data, metagenomic Kraken2/Bracken profiles detect thousands of species across the full tree of life, naturally producing much larger alpha values than amplicon studies (51).

**Table 3:** Alpha diversity indices calculated from bracken-estimated species-level abundance profiles of the gut microbiome of *A. ahngerianus* and *A. turkestanicus*.

Index	A. ahngerianus	A. turkestanicus
Shannon’s diversity (H’) Richness + evenness	4.046	3.363
Simpson’s index of diversity (1 − D) Dominance-corrected diversity	0.927	0.776
Fisher’s alpha (α) Log-series species richness	240.1	214.1
Berger-Parker index (d) Dominance of most abundant taxon	0.204	0.465
Total classified reads Input to diversity analysis	924,255	587,978

The bold values represent the total read counts for each gut microbiome sample.

### 3.4 Functional role of microbial communities

Functional annotation of the gut metagenomes revealed both shared and species-specific differences in the predicted metabolic potential of the microbial communities associated with *A. ahngerianus* and *A. turkestanicus*. In total, 3,562 uniquely identified genes were annotated from the gut microbiome of *A. ahngerianus*, while 3,626 uniquely identified genes were annotated from *A. turkestanicus*. These results indicate that the two termite species possess broadly comparable but not identical microbial functional profiles.

COG-based functional classification showed that both gut microbiomes were dominated by genes involved in translation, ribosomal structure and biogenesis, amino acid transport and metabolism, carbohydrate transport and metabolism, replication, recombination and repair, energy production and conversion, and signal transduction mechanisms. The presence of these categories suggests that both termite gut microbiomes contain metabolically active microbial communities capable of supporting digestion, nutrient cycling, and microbial population maintenance.

Although the observed differences in functional category proportions are modest, these trends suggest subtle functional variation between the two microbiomes, potentially influenced by host-specific ecological and dietary factors. Given that COG classification represents broad functional categories rather than specific metabolic pathways, these interpretations should be considered indicative of overall functional potential rather than definitive functional differences.

**Figure 5:**
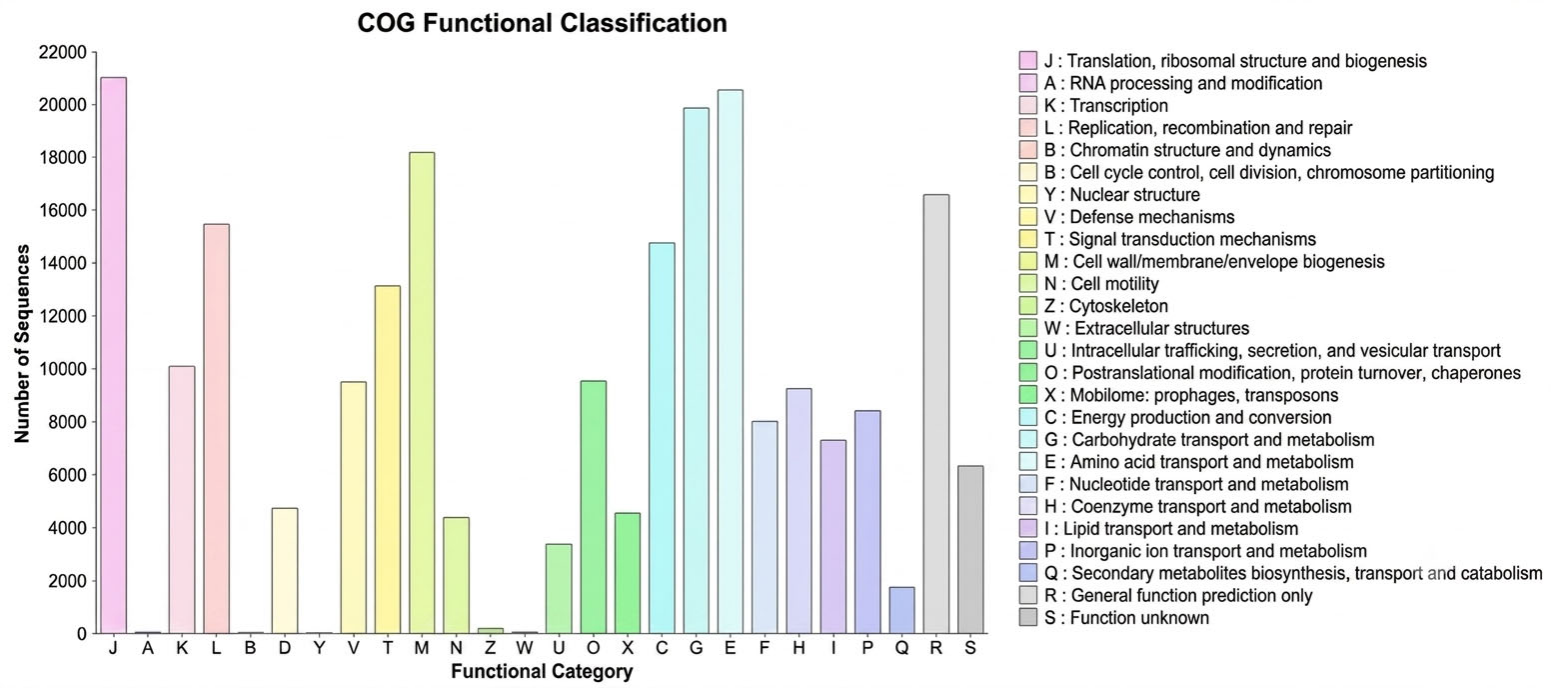
COG functional classification result for *A*. *ahngerianus.* Comparative COG functional profiles of gut microbiomes of *A. ahngerianus.* Bars show the relative abundance of major COG functional categories, including translation, amino acid transport and metabolism, carbohydrate transport and metabolism, replication and repair, energy production and conversion, signal transduction, and mobilome-related functions.

**Figure 6:**
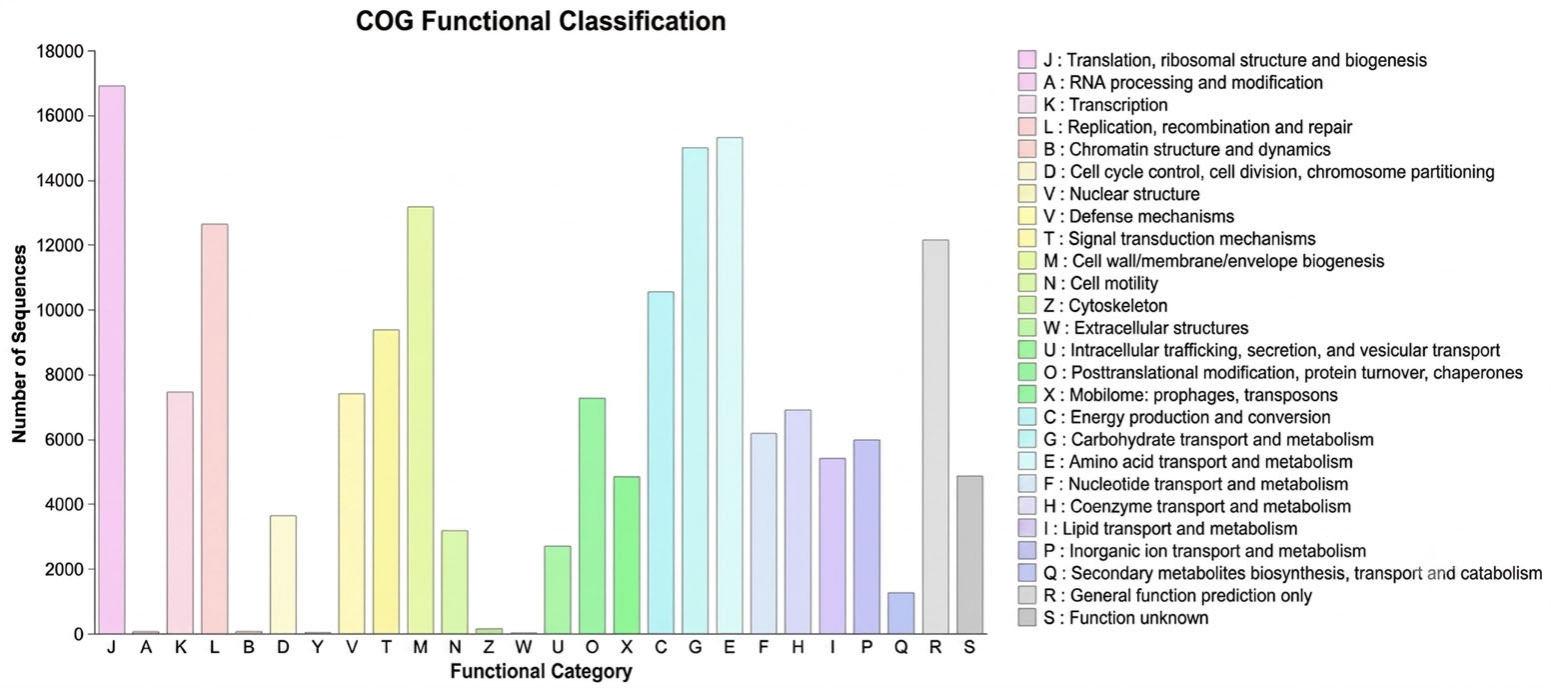
COG functional classification result for *A. turkestanicus.* Comparative COG functional profiles of gut microbiomes of *A. turkestanicus.* Bars show the relative abundance of major COG functional categories, including translation, amino acid transport and metabolism, carbohydrate transport and metabolism, replication and repair, energy production and conversion, signal transduction, and mobilome-related functions.

The functional profiles observed in both termite species can be linked to the dominant bacterial taxa identified in the metagenomic analysis. The enrichment of COG category G (carbohydrate transport and metabolism) in both species is consistent (**Figures 5**, **6**) with the high relative abundance of Pseudomonadota and Spirochaetota detected in both termites, as MAGs derived from *Pseudomonadota* (*Burkholderiales*, *Pseudomonadales*) encode oxygen-dependent enzymes that oxidize cellulose and modify lignin, representing lineages located at the hindgut wall (52). Bacteroidota, which were highly abundant in *A. ahngerianus*, have been implicated in cellulose and hemicellulose hydrolysis in lower termites as ectosymbionts of oxymonadid flagellates (32), suggesting that the elevated COG category G signal in this species may partly reflect the metabolic contribution of this phylum. The enrichment of COG category E (amino acid transport and metabolism) may be linked to the presence of Spirochaetota, as Spirochaetae in termite guts are involved in urea transport and metabolism and, together with Fibrobacteres, are capable of synthesising ten essential amino acids that animals cannot synthesize *de novo* (53). The higher representation of COG category X (mobilome: prophages and transposons) in *A. turkestanicus* compared to *A. ahngerianus* may reflect, at least at the level of the two colonies we studied, greater genomic plasticity in the microbial community of this species, potentially associated with adaptation to the more anthropogenically disturbed urban habitat from which it was collected (33).

## 4 Discussion

**Figure 7:**
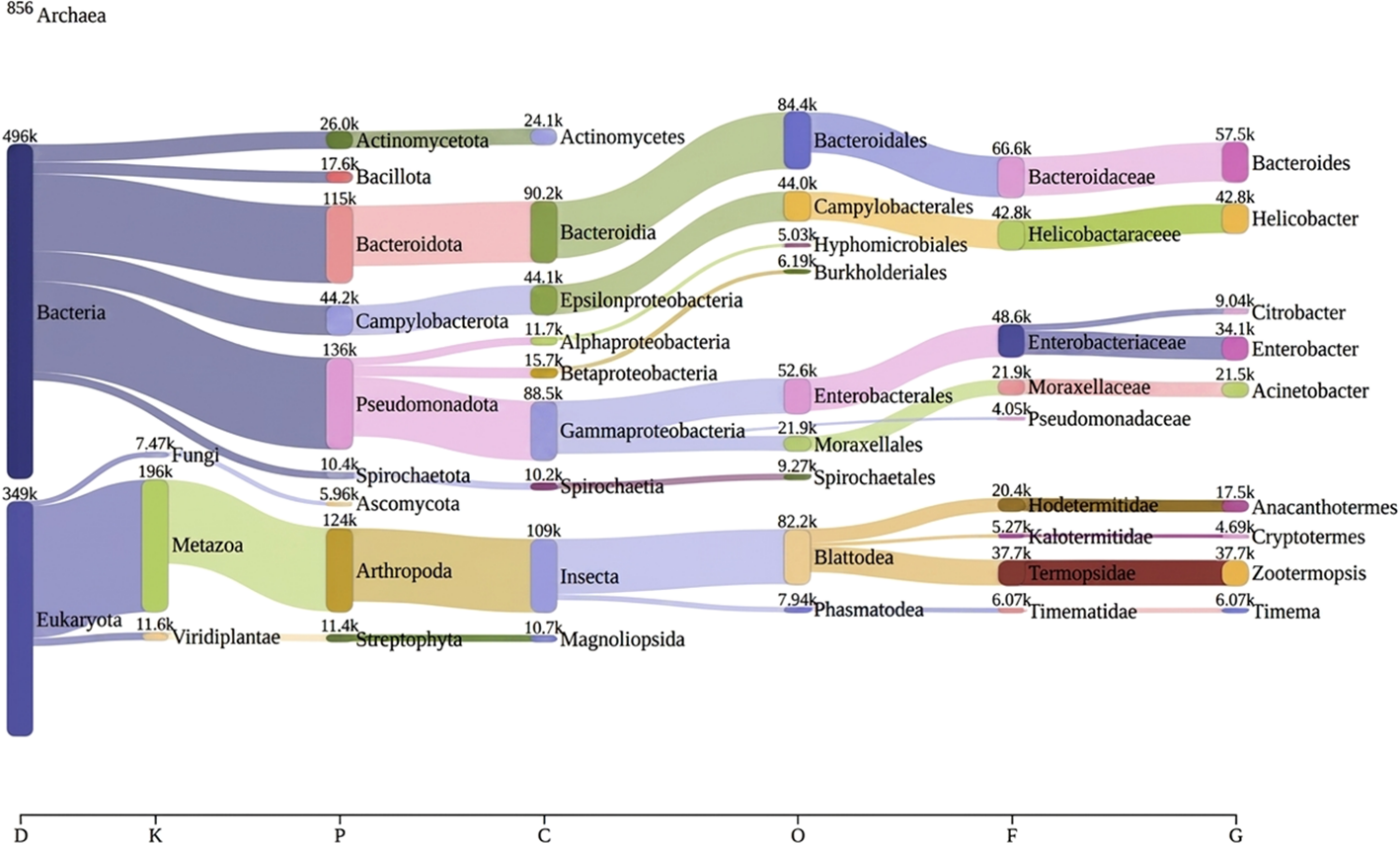
Taxonomic composition and hierarchical distribution of microbial communities across phylogenetic ranks (*A. ahngerianus*). The x-axis represents successive taxonomic ranks from left to right: D (Domain), K (Kingdom), P (Phylum), C (Class), O (Order), F (Family), and G (Genus). Flow widths are proportional to the number of assigned sequences (×10³) at each taxonomic level.

**Figure 8:**
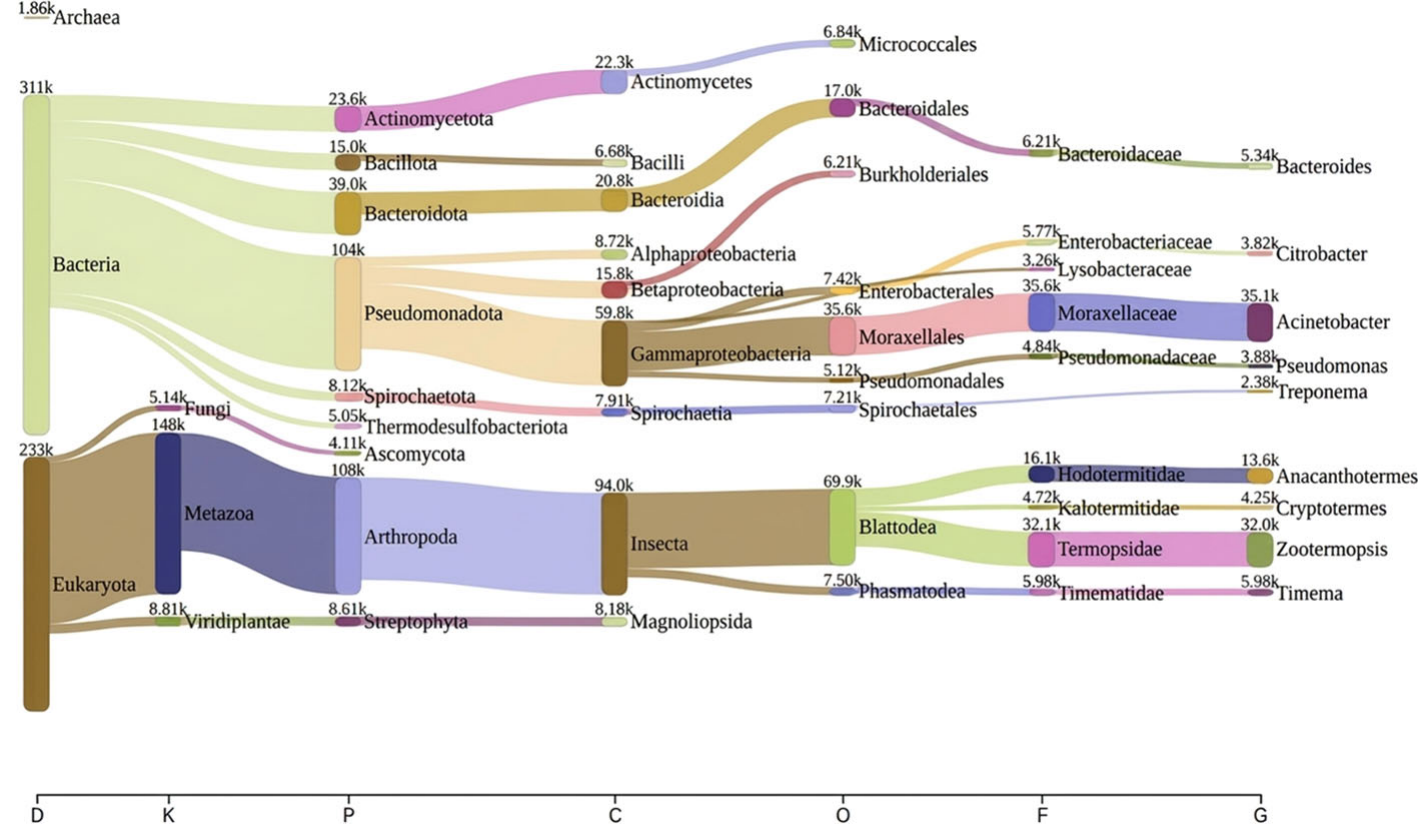
Taxonomic composition and hierarchical distribution of microbial communities across phylogenetic ranks (*A. turkestanicus*). The x-axis represents successive taxonomic ranks from left to right: D (Domain), K (Kingdom), P (Phylum), C (Class), O (Order), F (Family), and G (Genus). Flow widths are proportional to the number of assigned sequences (×10³) at each taxonomic level.

This study represents the first comparative metagenomic analysis of the gut microbiomes of the lower termites *A. turkestanicus* and *A. ahngerianus*. Although they are closely related, the two species harbor distinct microbial communities, probably reflecting adaptations to their respective habitats and diets, at least for the two colonies used for this study. Indeed, *A. turkestanicus* has been collected in urban areas while *A. ahngerianus* was collected in nature. These findings reinforce the view that environmental conditions and dietary ecology, rather than phylogeny alone, are central in shaping termite gut microbiomes. This statement should be confirmed by analyzing the gut microbiome of *A. turkestanicus* collected in nature and the one of *A. ahngerianus* in urban areas. At the kingdom level (**Figures 2**, **7**, **8**), the gut microbiome of both *A. ahngerianus* and *A. turkestanicus* was dominated by Bacteria, accounting for 53.66% and 52.98% of total reads, respectively. The near-identical proportions between the two species suggest a conserved bacterial community structure at the kingdom level, likely reflecting shared ecological roles and phylogenetic history. The composition and function of termite gut prokaryotic communities have been shown to be conserved since termites first appeared approximately 150 million years ago (32).

The bacterial community composition further underscores dietary specialization (**Figure 3**). The gut microbiomes of both *A. turkestanicus* and *A. ahngerianus* contained a shared microbial core composed mainly of bacterial phyla commonly associated with termite digestion, including Pseudomonadota, Bacteroidota, Actinomycetota, Bacillota, Spirochaetota, and Campylobacterota. These shared groups likely represent functionally important members of the *Anacanthotermes* gut ecosystem because they are associated with carbohydrate metabolism, fermentation, amino acid metabolism, energy production, and maintenance of gut microbial homeostasis. In lower termites, such bacteria act together with flagellated protists, particularly parabasalid and oxymonadid symbionts, to degrade cellulose and hemicellulose into fermentable products that can be used by the host. In addition, archaeal members, mainly Euryarchaeota, may contribute to hydrogen turnover and methanogenesis, while bacteriophages may regulate bacterial population dynamics within the gut. Therefore, the shared microbial community detected in both termite species can be interpreted as a conserved symbiotic core supporting lignocellulose digestion, nutrient acquisition, and physiological stability. In contrast, several taxa showed species-specific or allochthonous patterns. *A. ahngerianus* showed higher overall microbial diversity and contained unique eukaryotic genera such as *Rhinotermes*, and *Trichomitopsis*, whereas *A. turkestanicus* exclusively contained *Ruficoccus* among bacteria, like the eukaryotic genera *Noditermes* and *Dichotrachelus*. Higher microbial diversity in *A. ahngerianus* is consistent with previous studies showing that termites inhabiting natural environments and feeding on diverse plant substrates often harbor more complex gut microbial communities than species associated with anthropogenic habitats. For example, Mikaelyan et al. (54) demonstrated that diet is a major driver of gut bacterial diversity in termites, while Waidele et al. (16, 17) showed that ecological niche strongly influences both bacterial and protist community composition. The greater diversity observed in *A. ahngerianus* may therefore reflect differences in nesting environment, on feeding substrate or exposure to a wider range of environmental microorganisms and plant-derived substrates in natural desert ecosystems. However, because the present study used short-read shotgun metagenomic data and pooled samples, these taxonomic assignments should be interpreted cautiously, especially at species level. Therefore, we treated genus-level patterns as more reliable and interpreted allochthonous taxa as ecological indicators rather than experimentally confirmed functional symbionts.

*A. turkestanicus* was enriched in Pseudomonadota and Spirochaetota, which are putatively associated with cellulose hydrolysis and fermentation (33, 55). By contrast, *A. ahngerianus* showed higher relative abundances of Campylobacterota and Bacteroidota, phyla previously linked to secondary metabolism and polysaccharide fermentation (56, 57). These complementary strategies may indicate that gut microbiomes have diversified to optimize resource use under different ecological conditions. We hypothesize that variations in ecological conditions may drive shifts in the gut microbiome composition, thereby optimizing food degradation efficiency, similar to patterns observed in termites (54, 58) and in other animals [e.g. in ants (59): in birds (60)].

Genus-level comparisons (**Table 2**) revealed exclusive associations that may support species-specific adaptations. *A. ahngerianus* harbored a notably greater number of unique genera, all belonging to Eukaryota, including *Rhinotermes*, *Trichomitopsis*, and seven additional eukaryotic genera, suggesting a richer and more diverse eukaryotic community. Notably, *Anacanthotermes* belongs to the harvester termite family Hodotermitidae, and certain symbiont genera such as *Rostronympha*, *Spiromastigotes*, *Kirbynia*, and *Polymastigoides* are unique to *Anacanthotermes* hosts (34). In contrast, *A. turkestanicus* exclusively harbored *Ruficoccus* (Bacteria), *Noditermes*, and *Dichotrachelus* (Eukaryota). The higher number of unique eukaryotic genera in *A. ahngerianus* is consistent with its greater overall alpha diversity and may reflect differences in foraging microhabitat and soil exposure between the two collection sites.

Eukaryotic profiles differed in the relative abundance of taxonomic groups detected between the two species, at least in the two colonies we used (**Table 2**). Arthropoda-associated reads most likely originated from residual host DNA, as host-genome filtering was not possible due to the lack of a reference genome for the termite species that we are studying. In contrast, the detection of Chordata-associated reads was unexpected given the strictly lignocellulosic diet of *Anacanthotermes* species and may result from misclassification of conserved genomic regions by k-mer-based classifiers such as Kraken2, which are known to generate false-positive assignments across distantly related taxa in shotgun metagenomic datasets (61). These findings highlight the need for cautious interpretation of low-abundance eukaryotic signals in insect gut metagenomes.

Viruses comprised a minor but detectable fraction of the gut metagenome in both species, representing 0.37% of reads in *A. ahngerianus* and 0.39% in *A. turkestanicus* (**Table 1**), with no substantial difference between the two species. The phylum Uroviricota predominated in both species, accounting for more than 60% of total viral sequences (**Figure 3**). Termite gut viromes are dominated by temperate bacteriophages, with evidence suggesting a core virome comprised of bacteriophages infecting endosymbionts of gut protozoa, pointing to a quadripartite relationship between the termite, its symbiotic protozoa, their endosymbiotic bacteria, and associated bacteriophages (62). The consistent and near-identical viral fractions across both *Anacanthotermes* species may therefore reflect a conserved core virome linked to their shared protist and bacterial communities, rather than species-specific ecological pressures.

Archaea, though present at lower abundance, were dominated by methanogenic Euryarchaeota (**Figure 3**), consistent with previous reports of their role in hydrogen turnover and methane production in termite guts (63). The detection of haloarchaeal genera (e.g., Halogeometricum, Haloferax, Haloquadratum) is particularly noteworthy, as these taxa are rarely reported in termite guts. Their occurrence may reflect adaptation to the arid and saline soils of Central Asia. While these archaea are famous for living in salt lakes, they are also highly diverse in dry, salty soils where they survive extreme dehydration (64, 65). Termites likely pick up these microorganisms while foraging or building nests with local soil. Once inside the gut, these archaea are well-equipped to survive the unique chemical conditions and high alkalinity found in certain gut sections. This suggests that the termite gut acts as a protected micro-environment for specialized soil microbes, potentially assisting in the breakdown of organic matter under harsh conditions (66).

Nevertheless, certain methodological considerations should be noted. Due to constraints in resources, pooled gut samples from 15 workers per species were used to secure sufficient DNA yield for sequencing. While this approach limited the ability to assess intra-species variability, it nevertheless provided a broad overview of the core microbial community, which is critical for generating the first comparative baseline of *Anacanthotermes* gut microbiomes. Similarly, functional insights were derived from predictive annotations rather than experimental assays; while preliminary, such predictions are valuable for formulating testable hypotheses that can now be addressed through targeted functional validation. Finally, reliance on DNA-based metagenomics, although not capturing RNA viruses or transcriptionally active interactions, enabled the construction of a stable genomic inventory of the gut microbiota. Establishing this inventory represents a necessary foundation upon which future studies incorporating metatranscriptomics, metabolomics, and replicate sampling can build to achieve a deeper functional and ecological understanding. It is important to note that taxonomic assignments at the species level based on short-read metagenomic data (150 bp) should be interpreted with caution. Tools such as Kraken2 rely on k-mer matching against reference databases, which may lead to ambiguous classification among closely related taxa. Therefore, genus-level patterns are considered more reliable in this study.

Building on these findings and by keeping in mind the methodological constraints, the present work may be considered as the first step in the identification of the microbiome from these two termite species and our understanding of termite–microbiome interactions. The identification of distinct bacterial, fungal, viral, protozoan, and archaeal assemblages underscores how ecological context drives microbiome divergence even within a single termite genus. These insights extend termite symbiosis research by highlighting complementary metabolic strategies for lignocellulose degradation, nitrogen cycling, and microbial regulation, strengthening the concept of termites as holobionts. The detection of taxa with unique metabolic capacities, including nitrogen-fixing bacteria, potential ligninolytic fungi, and haloarchaea, points to microbial reservoirs that may underlie termite adaptation to arid habitats.

Future research should focus on high-resolution approaches to validate and extend these observations with more colonies and without any pooled samples. Metatranscriptomics and metabolomics would distinguish active from latent microbial functions, while recovery of metagenome-assembled genomes (MAGs) could assign functional potential to specific lineages. Replicate sampling across seasons, habitats, and developmental stages would clarify microbiome stability and variability. Experimental assays of candidate taxa, combined with dietary manipulations, would provide causal evidence for the ecological roles inferred here.

In conclusion, despite close phylogenetic relatedness, *A. turkestanicus* and *A. ahngerianus* in our study harbor distinct gut microbial assemblages reflecting divergent ecological adaptations. By considering the methodological limitations of our study, workers in *A. turkestanicus* exhibited stronger bacterial dominance associated with lignocellulose degradation and nitrogen cycling, while workers in *A. ahngerianus* maintained a more balanced representation of bacteria, fungi, and viruses, suggesting alternative digestive strategies. Functional annotation suggested that the gut microbial communities of the studied termites may possess metabolic potential related to carbohydrate metabolism, amino acid transport, and microbial defense mechanisms. These predicted functions are relevant to termite nutrition because gut microorganisms are expected to contribute to the processing of lignocellulosic diets and to support host adaptation to nutrient-limited environments. However, these functions were inferred from metagenomic data and were not experimentally verified in the present study. Moreover, the pooled workers and the limited number of colonies used do not allow us to generalize our findings. Therefore, the results should be interpreted as predicted functional potential rather than confirmed microbial activity. Collectively, these findings provide a genomic baseline for understanding possible microbiome-mediated ecological adaptation in desert termites. Future studies using enzyme activity assays, metatranscriptomics, metaproteomics, and metabolomics are required to validate the functional roles of specific gut microorganisms and to evaluate their potential relevance for microbiome-based bioconversion, bioenergy, and sustainable termite management.

## Data Availability

The datasets presented in this study can be found in online repositories. The names of the repository/repositories and accession number(s) can be found in the article/Supplementary Material.
